# Divergent Expression of *SPARC*, *SPARC-L*, and *SCPP* Genes During Jawed Vertebrate Cartilage Mineralization

**DOI:** 10.3389/fgene.2021.788346

**Published:** 2021-11-25

**Authors:** Adrian Romero, Nicolas Leurs, David Muñoz, Mélanie Debiais-Thibaud, Sylvain Marcellini

**Affiliations:** ^1^ Laboratory of Development and Evolution (LADE), University of Concepción, Concepción, Chile; ^2^ Institut des Sciences de l’Evolution de Montpellier, ISEM, Univ Montpellier, CNRS, IRD, EPHE, Montpellier, France

**Keywords:** *SPARC*, *SPARC-L*, *SCPP*, cartilage mineralization, *Xenopus tropicalis*, *Scyliorhinus canicula*, vertebrate evolution

## Abstract

While cartilage is an ancient tissue found both in protostomes and deuterostomes, its mineralization evolved more recently, within the vertebrate lineage. *SPARC*, *SPARC-L*, and the *SCPP* members (Secretory Calcium-binding PhosphoProtein genes which evolved from *SPARC-L*) are major players of dentine and bone mineralization, but their involvement in the emergence of the vertebrate mineralized cartilage remains unclear. We performed *in situ* hybridization on mineralizing cartilaginous skeletal elements of the frog *Xenopus tropicalis* (*Xt*) and the shark *Scyliorhinus canicula* (*Sc*) to examine the expression of *SPARC* (present in both species), *SPARC-L* (present in *Sc* only) and the *SCPP* members (present in *Xt* only). We show that while mineralizing cartilage expresses *SPARC* (but not *SPARC-L*) in *Sc*, it expresses the *SCPP* genes (but not *SPARC*) in *Xt*, and propose two possible evolutionary scenarios to explain these opposite expression patterns. In spite of these genetic divergences, our data draw the attention on an overlooked and evolutionarily conserved peripheral cartilage subdomain expressing *SPARC* or the *SCPP* genes and exhibiting a high propensity to mineralize.

## Introduction

The evolution of a mineralized skeleton occurred in early vertebrates, in a variety of tissues including superficial dermal scales and teeth, together with internal cartilages, and perichondral bones (Ørvig, 1951; Donoghue and Sansom, 2002). In the internal skeleton, several cell types are associated with biomineralization, and the most studied cell model in mammalian organisms is the osteoblast active in the endochondral ossification process (Long and Ornitz, 2013). These osteoblasts are derived from periosteal tissues or from hypertrophic transdifferentiated chondrocytes (Tsang et al., 2015). The process of endochondral ossification, or replacement of cartilage matrix by bone marrow and bone trabeculae, is absent from chondrichthyans and has long been thought to be a derived feature specific to osteichthyans (reviewed in Donoghue and Sansom, 2002; Hirasawa and Kuratani, 2015), although recent paleontological data has challenged this view (Brazeau et al., 2020). Also known to mineralize their matrix are the chondrocytes, not only at the ossification front of endochondral bone growth (in the case of hyaline cartilage), but also in stable forms of mineralized cartilage such as fibrocartilages and other forms of cartilage displaying striking similarities to bony tissues (Beresford, 1981; Dyment et al., 2015; Paul et al., 2016; Pears et al., 2020; Berio et al., 2021). Even though both perichondral bones and cartilaginous tissues displayed mineralization in the earliest forms of mineralized internal skeletons (Ørvig, 1951; Min and Janvier, 1998; Donoghue et al., 2006; Johanson et al., 2010, 2012; Pears et al., 2020), mineralizing cartilages have been understudied from a genetic and evolutionary perspective in extant vertebrates. A better understanding of the genetic underpinning of the mineralizing chondrocytes is therefore necessary to understand the early steps of the evolution of endoskeletal mineralization in vertebrates.

**FIGURE 1:**
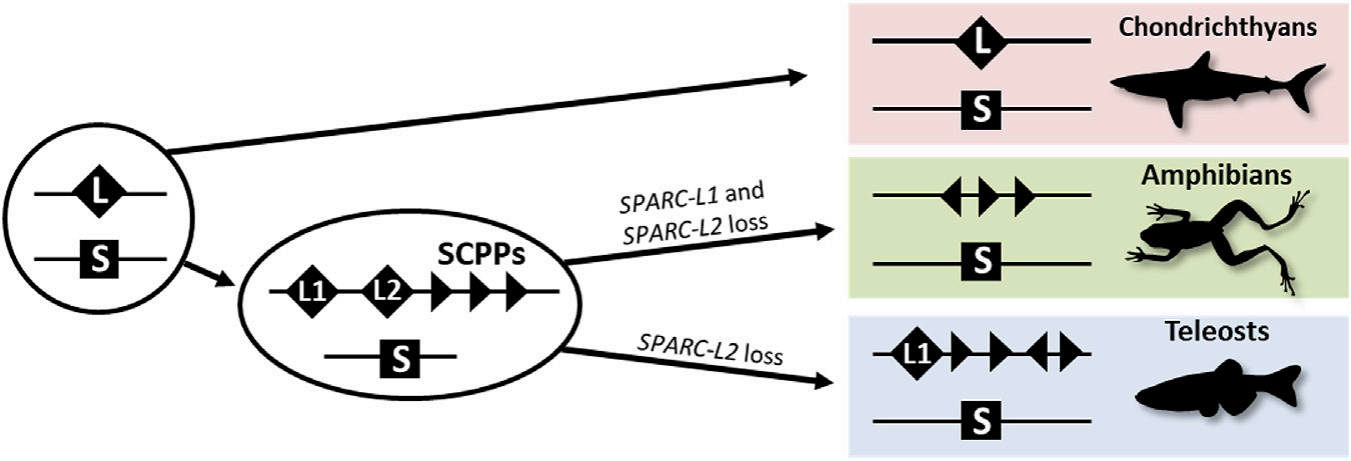
A simplified evolutionary scenario for the *SPARC*/*SPARC-L*/*SCPP* family. Vertebrate-specific whole genome duplications produced the ancestral *SPARC* (S) and *SPARC-L* (L) paralogues. These loci were not overtly altered in the chondrichthyan lineage as both genes are clearly identifiable in sharks. In the osteichthyan lineage, local duplications at the *SPARC-L* locus produced *SPARC-L1* (L1), *SPARC-L2* (L2) and the *SCPP* members (triangles). Triangles in different orientations symbolize the fact that *SCPP* genes are subject to independent local duplications events and a high rate of evolutionary divergence, hindering homology relationships. *SPARC-L2* was independently lost in tetrapods and teleosts and *SPARC-L1* was also lost in amphibians. See text for details and references.

The evolution of vertebrate endoskeletal mineralization has been discussed in the light of the two rounds of whole-genome duplication (2Rs). These duplications occurred before the diversification of extant jawed vertebrates (Nakatani et al., 2021) and generated gene families with diverging gene functions which may have produced the genetic toolkit required for the cellular ability to mineralize an extracellular matrix (Zhang and Cohn, 2008). The evolution of the *SPARC*/*SPARC-L*/*SCPP* gene family has been of great interest in this perspective (Kawasaki and Weiss, 2003; Kawasaki et al., 2005; Kawasaki, 2009; Bertrand et al., 2013; Enault et al., 2018), and is summarized in Figure 1. *SPARC-L* and *SPARC* are two paralogues having originated from the 2Rs (Kawasaki and Weiss, 2003; Kawasaki et al., 2005; Kawasaki, 2009; Bertrand et al., 2013; Enault et al., 2018). In bony fishes, independent local duplications at the *SPARC-L* locus generated *SPARC-L1* and *SPARC-L2* and a variable number of tandemly located genes coding for Secretory Calcium-binding PhosphoProteins (SCPPs) that have evolved rapidly since their origin (see Supplementary Figure S1 and Kawasaki and Weiss, 2003; Kawasaki et al., 2005; Enault et al., 2018). Hence outside of amniotes, homology relationships between *SCPP* duplicates are obscured by independent gene gains and losses together with a high rate of sequence divergence (Kawasaki, 2009). No *SCPP* genes have been identified in cartilaginous fish genomes, making the chondrichthyan *SPARC-L* gene the single orthologue to all *SCPP* genes of bony vertebrates (see Figure 1, Supplementary Figure S1 and Ryll et al., 2014; Venkatesh et al., 2014; Enault et al., 2018).

The *SPARC* gene (Secreted Protein Acidic and Rich in Cysteine, formerly coined *Osteonectin*) encodes a matricellular protein which is one of the most abundant non-collagenous matrix proteins in mammalian and teleost bone (Schreiweis et al., 2007; Kessels et al., 2014). Secreted by osteoblasts, the SPARC protein functions in mineralized tissues by binding both collagen fibrils and calcium, but also by signaling to bone cells (reviewed by Rosset and Bradshaw, 2016). In osteichthyans, the expression of *SPARC* is evolutionary conserved in osteoblasts as well as in odontoblasts (Holland et al., 1987; Li et al., 2009; Espinoza et al., 2010; Enault et al., 2018). In chondrichthyans having secondarily lost the bone tissue (and the osteoblast cell type), *SPARC* is highly expressed in odontoblasts (Enault et al., 2018). The single *SPARC-L* gene in cartilaginous fishes is expressed in enameloid secreting cells in teeth and scales of the catshark *Scyliorhinus canicula* (Enault et al., 2018). In osteichthyans it seems that *SPARC-L1* and *SPARC-L2* are not specifically expressed nor functionally required in the skeleton (McKinnon et al., 2000; Bertrand et al., 2013). In addition, *SPARC-L2* was independently lost in tetrapods and teleosts, and *SPARC-L1* was also lost in amphibians (see Figure 1 and Kawasaki et al., 2007; Bertrand et al., 2013; Enault et al., 2018), suggesting that these two genes are functionally dispensable. Rather, in osteichthyans, *SCPP* family members are key players of skeletal mineralization. Within amniote *SCPP* genes, *Bone sialoprotein* (*BSP*), *Osteopontin* (*OPN* or *SPP1*) and *Dentin matrix protein 1* (*DMP1*) are strongly expressed by osteoblasts and their protein products are stored in the mineral phase of bone tissue (Ustriyana et al., 2021). Most members of this family are also expressed and functional during tooth development in mammals (either in the production of enamel or/and dentin, reviewed by Nikoloudaki, 2021). In the clawed frog *Xenopus tropicalis* and the zebrafish *Danio rerio* the expression of distinct *SCPP* members has been reported in ameloblasts, odontoblasts, and osteoblasts (Kawasaki et al., 2005; Kawasaki, 2009; Espinoza et al., 2010; Enault et al., 2018). Overall, our knowledge of the evolution of the expression of *SPARC*, *SPARC-L* and the *SCPP* members during cartilage mineralization remains limited, and, in this study, we examined the expression of these genes during endoskeletal development in *Xenopus tropicalis* and *Scyliorhinus canicula*.

## Methods

### Specimens, Histological Staining and Cryo-Sections

Lesser spotted catshark (*Scyliorhinus canicula*) embryos were maintained at 17°C at the University of Montpellier, France, until they reached development stage 32 (Ballard et al., 1993; Maxwell et al., 2008). Embryos were taken out of their eggshell, anesthetized and subsequently euthanized by overdose of MS-222 (Sigma) following European animal-care specifications. As substantial growth occurs during stage 32, each individual was measured before fixation and classified into early, intermediate and late stages whose body length measured respectively 5.3, 6.6, and 8.5 cm for histological analyses, and respectively 5.0, 6.3, and 7.9 cm for the Alizarin red S and *in situ* hybridization procedures. Abdominal vertebral portions were fixed 48 h in PFA 4% in PBS 1× at 4°C and were subsequently transferred in ethanol and stored at − 20°C until needed.

Adult *Xenopus tropicalis* were maintained following standard protocols established for this species, at the University of Concepcion. Embryos and tadpoles were raised after natural mating and staged according to the Nieuwkoop and Faber developmental table (Nieuwkoop and Faber, 1967). Anesthesia of tadpoles was performed with a MS-222 (Sigma) solution at 2 mg/ml and each specimen was subsequently decapitated in agreement with international bioethical recommendations (Close et al., 1996; Ramlochansingh et al., 2014).

Dissected organs of both species were embedded in paraffin to generate 7 μm-thick histological sections that were stained with standard protocols [eosin, hematoxylin and safran reaction for catshark (RHEM platform at IRCM, Montpellier); hematoxylin and chromotrope 2R (C3143 Sigma) for frog sections]. The von Kossa protocol was used on paraffin sections of *Xenopus tropicalis* to detect calcium on tissue sections (#10241, Diapath, Italy) following the manufacturer’s instruction. Briefly, the von Kossa method is based on the transformation of calcium ions, bound to phosphates, into silver ions brought by a solution of silver nitrate.

Spotted catshark alizarin red S and *in situ* hybridizations were performed on serial, 14 μm thick cryostat sections, cut transversal in the body trunk, at the level of the pectoral fins. Parts of the specimens that were not used for this study were conserved in ethanol at − 20°C for further studies on gene expression. Alizarin red S staining was used to detect calcium deposits with a single bath of 0.05% Alizarin Red S (Sigma) in KOH 0.5%, 5 min, before mounting in Mowiol. All slides generated with catshark samples were scanned on a Hamamatsu nanozoomer.

### *In situ* Hybridizations

All probes and *in situ* hybridization procedures used here with *Scyliorhinus canicula* and *Xenopus tropicalis* were previously described (Espinoza et al., 2010; Enault et al., 2018), except for the frog *SCPPA2* gene (GenBank EU642617) for which a 968 bp product was amplified and cloned into pBluescript using the following primers (5′ to 3′) Forward- GAG TCA TAC TAC AGG ACC TGC, Reverse-CAT GCA ACT CAG CCA AAG CT.

## Results

### *SPARC* and *SPARC-L* Expression in the Development of Vertebrae in the Lesser Spotted Catshark *Scyliorhinus canicula*

**FIGURE 2:**
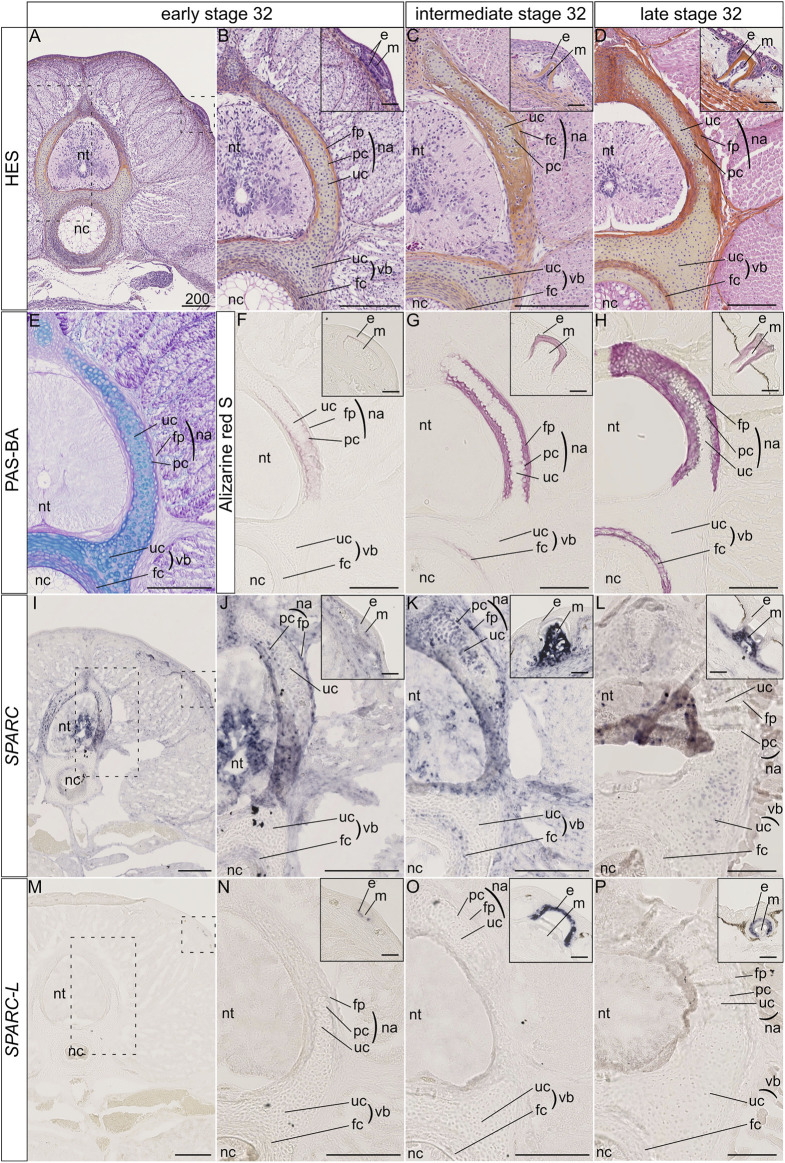
Histology, mineralization dynamics and *SPARC* and *SPARC-L* expression pattern in the vertebrae and scales of the small-spotted catshark *Scyliorhinus canicula*. **(A–D)** Hematoxylin-Eosin Safran (HES) histological staining on transverse sections at the level of thoracic vertebrae: A, general view with location of the neural tube (nt) and notochord (nc); B-D, closer views on vertebral tissues as boxed in A, with identification of the fibrous perichondrium (fp), unmineralized cartilage (uc) and peripheral chondrocytes (pc) of the neural arch (na), as well as the unmineralized cartilage (uc) and fibrous cartilage (fc) of the vertebral body (vb); insets in B-D, closer view of the dorsal scales as boxed in A, indicating the location of the epithelial (e) and mesenchymal (m) compartments of scale buds. Stage 32 embryos were staged according to their total length into “early,” “intermediate,” and “late” categories as described in the Material and Method section. **(E)** Periodic Acid Schiff-Alcian Blue (PAS-BA) histological staining of a section consecutive to A: BA (blue) stains the acid glycosaminoglycans of the hyaline cartilage and PAS (pink) stains the fibrous content of the perichondrium. **(F–H)** Alizarin red S staining locates calcium deposits in mineralizing matrices [of the peripheral chondrocytes (pc), fibrous perichondrium (fp) or fibrous cartilage (fc), and scale enameloid/dentin], in embryos of similar total length as A-D. **(I–L)** *SPARC* gene expression patterns, for sections that are consecutive to those shown in **(F–H)** respectively. **(M–P)** *SPARC-L* gene expression patterns for sections that are consecutive to those shown in **(F–H)** respectively. Scales represent 200 μm, except in scale insets where they represent 50 µm.

The catshark vertebral tissue mineralizes at the level of the neural arches and of the vertebral body (Enault et al., 2015). In the neural arches, mineralization occurs at two juxtaposed sites: the matrix of the most peripheral chondrocytes and the fibrous perichondrial tissue surrounding each neural arch (Figures 2A–H). In the neural arch peripheral cartilage, a faint Alizarin red-positive signal is observable in early stage 32 embryos (Figures 2A,B,E,F), and becomes more intense in intermediate and late stage 32 embryos (Figures 2C,D,G,H). Note that neural arch mineralization never extends to the center of the cartilaginous scaffold (Figures 2A–H and Berio et al., 2021). In addition, the fibrous perichondrial tissue surrounding each neural arch displays a robust mineralization in intermediate and late stage 32 embryos. In the vertebral body, a mineralization ring appears in the cartilage surrounding the notochord of intermediate stage 32 embryos and becomes more mineralized in late stage 32 embryos (Figures 2F–H).

The expression of the *SPARC* gene was detected in the neural tube and several connective tissues such as the dermis and perimysium at all tested developmental stages (Figures 2I–L). We report three major sites of *SPARC* expression in the *Sc* developing vertebrae: the neural arch chondrocytes, the neural arch fibrous perichondrium, and the vertebral body. In the neural arches of early stage 32 embryos, *SPARC* expression localizes to peripheral chondrocytes (*i.e.*, specifically to the mineralizing cartilage) and to the cells of the fibrous perichondrium (Figures 2I,J). In intermediate stage 32 embryos *SPARC* expression extends to most chondrocytes of the neural arches (Figure 2K). Cells of the mineralized fibrous perichondrial tissue surrounding the neural arches also express *SPARC* in intermediate and late stage 32 embryos (Figures 2J,K). In the vertebral body from early and intermediate stage 32 embryos, *SPARC* is detected as a ring of expression in chondrocytes surrounding the notochord (Figures 2I–K). Our results on late stage 32 embryos show little gene expression of *SPARC* in the vertebral tissues, as only a faint signal was observed in some chondrocytes, (Figure 2L), revealing that the expression of this gene is dynamic and transient in relation to the mineralization processes. We had previously shown that *SPARC* is expressed in developing scales (Enault et al., 2018), and the expected signal in odontoblasts presents on the same section strongly argues against a possible technical problem for the detection method in late stage 32 embryos (Figure 2L, inset).

On the other hand, the expression of *SPARC-L* could not be detected in any endoskeletal tissues, while its expression in the epithelium (*i.e.*, the ameloblast layer) of developing and mineralized scales was observable at all stages (Figures 2M–P), as expected (Enault et al., 2018).

### *SPARC* and *SCPPs* Expression in the Development of Limbs and Vertebrae in the Western Clawed Frog *Xenopus tropicalis*

**FIGURE 3:**
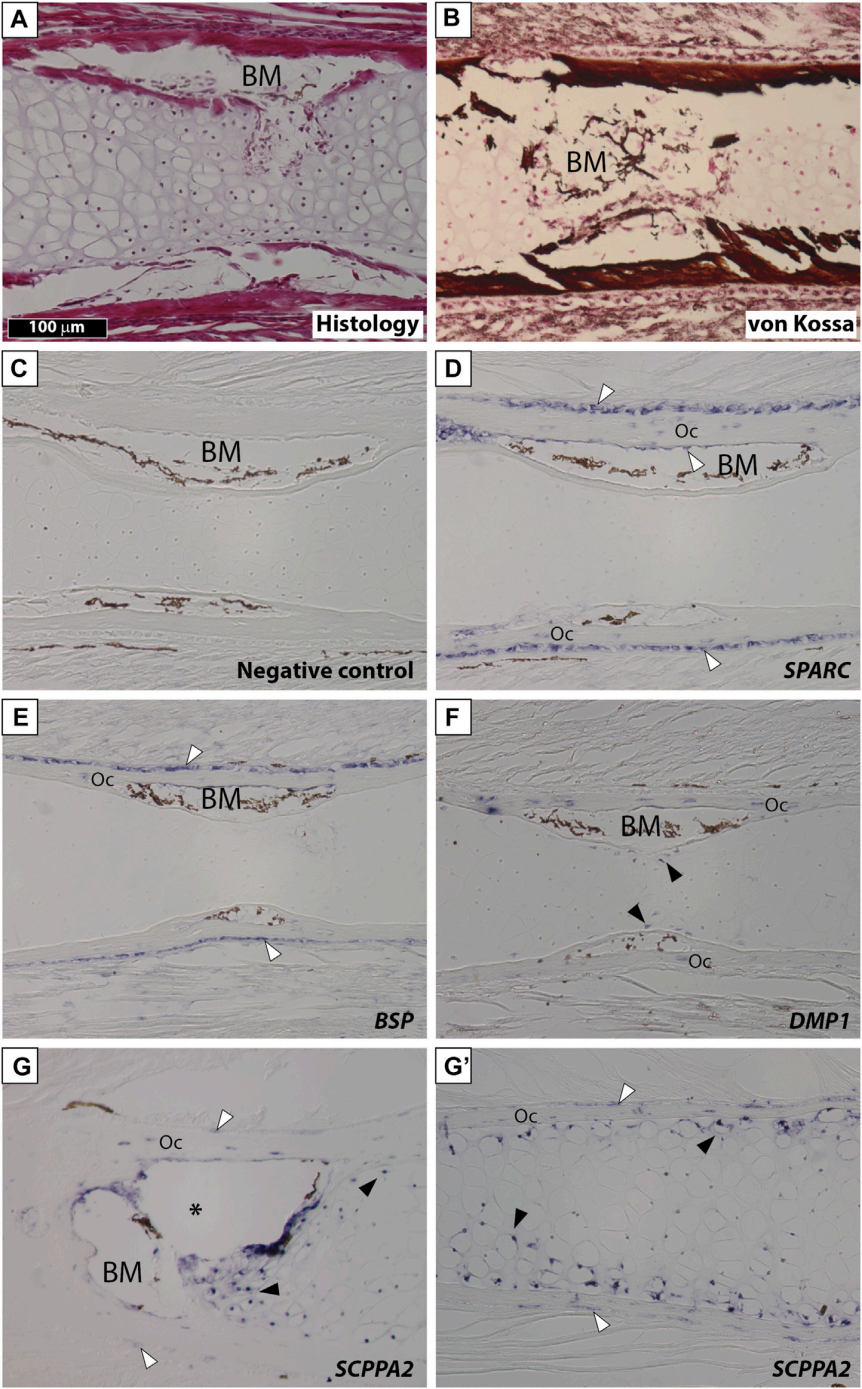
*SPARC* and *SCPP* gene expression in *Xenopus tropicalis* stage NF58 hindlimbs. Longitudinal sections of *Xenopus tropicalis* stage NF58 femoral bones were subjected to Hematoxylin-Eosin staining **(A)**, von Kossa staining **(B)**, or *in situ* hybridization using a negative control [*SPARC* sense probe, **(C)**] or an antisense probe for *SPARC* **(D)**, *BSP* **(E)**, *DMP1* **(F)**, and *SCPPA2* **(G, G′)**. Pictures show the diaphysis in **(A–G)** and the epiphysis in **(G′)**. White and black arrowheads show *in situ* hybridization signal in osteoblasts and chondrocytes, respectively. Abbreviations: BM, bone marrow, Oc osteocytes showing *in situ* hybridization signal. Scale bar in **(A)** represents 100 µm and applies to all panels. The asterisk shows an artifact due to the cartilage which teared apart and contracted in this region of the section.

We examined gene expression in NF58 *Xt* limbs because at this stage hypertrophic cartilage is in its most mature state and becomes eliminated and replaced by bone marrow at the diaphysis (Figure 3A). von Kossa staining showed an intense signal in the bone matrix and revealed that the *Xt* hypertrophic cartilage does not mineralize at the diaphysis of NF58 femoral bones (Figure 3B). *SPARC* transcripts were specifically detected in osteocytes and in osteoblasts of the periosteum and endosteum, but not in the cartilage (Figures 3C,D). A similar situation was observed for *BSP* (Figure 3E). *DMP1* was detected in osteocytes as well as in some chondrocytes of the diaphysis (Figure 3F). Transcripts from the *SCPPA2* gene were detected in osteocytes and some osteoblasts, and in many chondrocytes located at the cartilage to bone marrow transition and in the vicinity of the bone matrix of the diaphysis (Figure 3G) and of the epiphysis (Figure 3G′).

**FIGURE 4:**
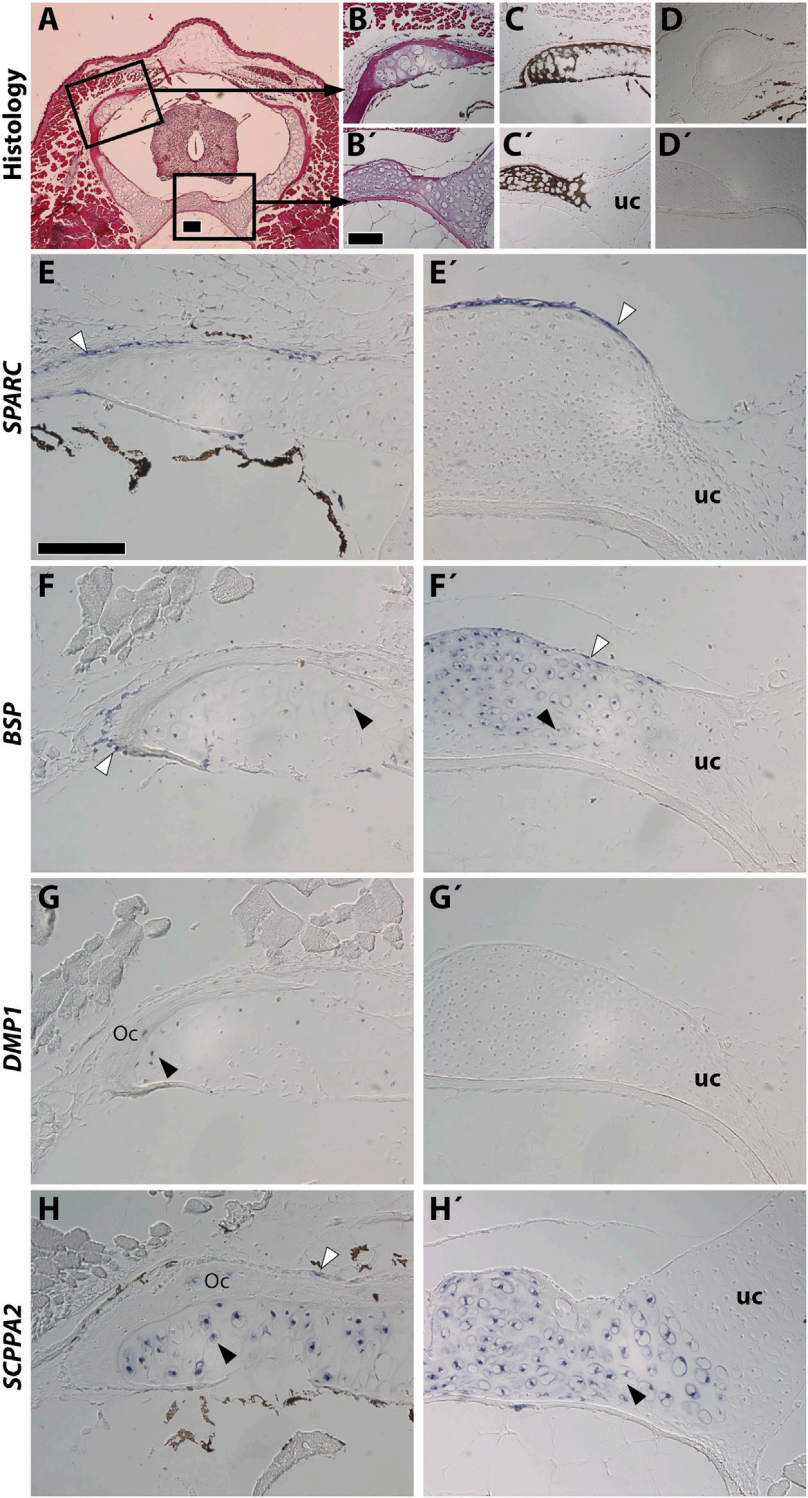
*SPARC* and *SCPP* gene expression in *Xenopus tropicalis* stage NF58 vertebrae. Transversal sections of *Xenopus tropicalis* stage NF58 vertebrae were subjected to Hematoxylin-Eosin staining **(A,B,B′)**, von Kossa staining **(C,C′)**, or *in situ* hybridization using a negative control [*SPARC* sense probe, **(D,D′)**] or an antisense probe for *SPARC* **(E,E′)**, *BSP* **(F,F′)**, *DMP1* **(G, G′)**, and *SCPPA2* **(H,H′)**. Pictures are orientated with dorsal to the top and show the whole vertebrae **(A)**, the neural arch **(B–H)** or the vertebral body **(B′–H′)**. White and black arrowheads show *in situ* hybridization signal in osteoblasts and chondrocytes, respectively. Abbreviations: uc, unmineralized cartilage, Oc osteocytes showing *in situ* hybridization signal. Scale bars: **(A)**, 100 μm; **(B)**, 100 µm in **(B–D′)**; **(E)**, 50 µm in **(E–H′)**.

Stage NF58 vertebrae (Figure 4A) were subjected to von Kossa staining, revealing cartilage mineralization in the dorsal region of the neural arches (Figures 4B,C), as well as in the ventral region located between the neural tube and the notochord (Figures 4B′,C′), in agreement with previous observations performed with Alizarin red S (Enault et al., 2015). We found that each of the examined genes displays a distinctive expression pattern. *SPARC* is specifically expressed in osteoblasts of the dorsal neural arches and of the ventral region, but not in chondrocytes (Figures 4D,D′,E,E′). *BSP* is expressed in osteoblasts and chondrocytes of both regions, albeit its expression is much stronger in the cartilage of the ventral vertebrae (Figures 4F,F′). *DMP1* is expressed in osteocytes and in chondrocytes located close to the bone matrix of the dorsal neural arch, but is not expressed in the ventral vertebra at this stage (Figures 4G,G′). *SCPPA2* is moderately expressed in some osteocytes and osteoblasts of the dorsal neural arch, and very strongly in chondrocytes of the mineralizing cartilage of both vertebral regions (Figures 4H,H′).

## Discussion

Our findings in *Xt* reveal an evolutionary conservation of the cartilaginous expression of the *SCPP* genes in tetrapods. Indeed, similarly to the situation in *Xt*, *SPARC* is not expressed in mouse chondrocytes (Holland et al., 1987). Rather, *SCPP* genes such as *DMP1* and *BSP* are expressed and required for mouse cartilage development (Chen et al., 1991; Ye et al., 2005; Bouleftour et al., 2014; Fujikawa et al., 2015). As indicated by other studies (Yagami et al., 1999; Bandyopadhyay et al., 2008), gene expression in cartilaginous elements can be subdivided in two distinct domains which we will use to discuss our results. First, *SCPP* genes become activated at late stages of hypertrophy, when the cartilage matrix becomes replaced by bone marrow at the mammalian diaphysis (Chen et al., 1991; Fujikawa et al., 2015). Likewise, in *Xt*, *DMP1* is exclusively expressed at the diaphysis (Figure 3F), and *SCPPA2* exhibits a much stronger expression at the diaphysis than the epiphysis region (Figures 3G,G′). A similar situation is observed at the level of the *Xt* vertebrae, where the expression of *SCPP* genes tightly correlates with cartilage maturation and mineralization in the neural arch (for *BSP*, *DMP1*, and *SCPPA2*) as well as in the ventral vertebral region (for *BSP* and *SCPPA2*). Second, the *SCPP* genes harbor a stronger expression in the non-mineralized peripheral cartilage, as observed in mouse for *osteopontin* (Heilig et al., 2016) and *DMP1* (Fujikawa et al., 2015). This situation is similar to the expression of *Xt SSCP* genes in chondrocytes located in the vicinity of the bone matrix in long bones and vertebrae (Figures 3, 4). Such a peripheral cartilage domain expresses specific genes, as reported in chick (Bandyopadhyay et al., 2008), and undergoes ectopic mineralization in mutant mouse animals for the *Mgp* (Marulanda et al., 2017) and *Trps1* (Napierala et al., 2008) genes. In summary, *SCPP* genes from frog and mouse are expressed in the mature cartilage of the diaphysis and neural arches, as well as in peripherally located chondrocytes.

Available expression analyses did not report any cartilaginous expression for *SCPP* genes in teleosts (Kawasaki et al., 2005; Kawasaki, 2009; Weigele et al., 2015). Rather, the expression of the *SPARC* gene has been associated to cartilage development in zebrafish, gilthead seabream and the cichlid mouth breeder (Estevao et al., 2005; Redruello et al., 2005; Rotllant et al., 2008; Estevao et al., 2011; Weigele et al., 2015), albeit not in medaka, at least at the examined stages (Renn et al., 2006). Hence the reported cartilaginous expression patterns in teleosts (*SPARC* positive and *SCPP* negative) are opposite to the tetrapod situation (*SPARC* negative and *SCPP* positive), which might be related to drastic difference in the mode of endochondral ossification between these two groups (Cervantes-Diaz et al., 2017). In this respect, our data in the chondrichthyan representative *Sc* is instrumental to understand the evolution of the expression of these genes in the jawed vertebrate endoskeleton.

As no *SCPP* genes have been reported in chondrichthyan genomes to date, we focused on the evolutionarily related gene *SPARC-L* (Kawasaki and Weiss, 2003; Bertrand et al., 2013; Venkatesh et al., 2014; Enault et al., 2018). Our finding that *Sc SPARC-L* is not expressed in the vertebral cartilage is further confirmed by the robust and expected *Sc SPARC-L* expression in the odontodes present on the same sections and serving as convenient internal positive controls (Enault et al., 2018). By contrast, *SPARC* expression clearly co-localizes with sites of vertebral mineralization. In the neural arches of early stage 32 embryos, *SPARC* is restricted to the mineralizing peripheral cartilage matrix, thereby paralleling the expression of *SCPP* genes in frog (Figures 2, 4) and mouse (Fujikawa et al., 2015; Heilig et al., 2016). Hence, we uncover a novel evolutionarily conserved cartilage domain, as defined by peripherally located chondrocytes expressing *SPARC* in chondrichthyans and the *SCPP* genes in tetrapods. One difference is that this domain mineralizes in chondricthyans, but not in tetrapods. We propose that dosage variations between pro- and anti-mineralizing proteins might explain evolutionary differences between vertebrate lineages, as might be the case for instance for MGP which is a well-conserved cartilage mineralization inhibitor (Yagami et al., 1999; Espinoza et al., 2010; Viegas et al., 2013; Marulanda et al., 2017; Leurs et al., 2021). By examining the centrum of *Sc* specimens from different developmental stages we show that a ring of *SPARC* expression is already present in Alizarin red-negative early stage 32 embryos, suggesting that the presence of the *SPARC* protein plays a crucial role in the initiation of mineralization. The functional interaction between the mammalian *SPARC* and collagen 1 proteins is important for mineralization (Termine et al., 1981). However, as the shark chondrocytes embedded within a mineralizing cartilage matrix neither expresses *collagen 1a1* nor *collagen 1a2* (Enault et al., 2015), *Sc SPARC* function might be related to other aspects of matrix mineralization, such as its interaction with calcium and hydroxyapatite crystals (Engel et al., 1987; Maurer et al., 1995; Fujisawa et al., 1996).

**FIGURE 5:**
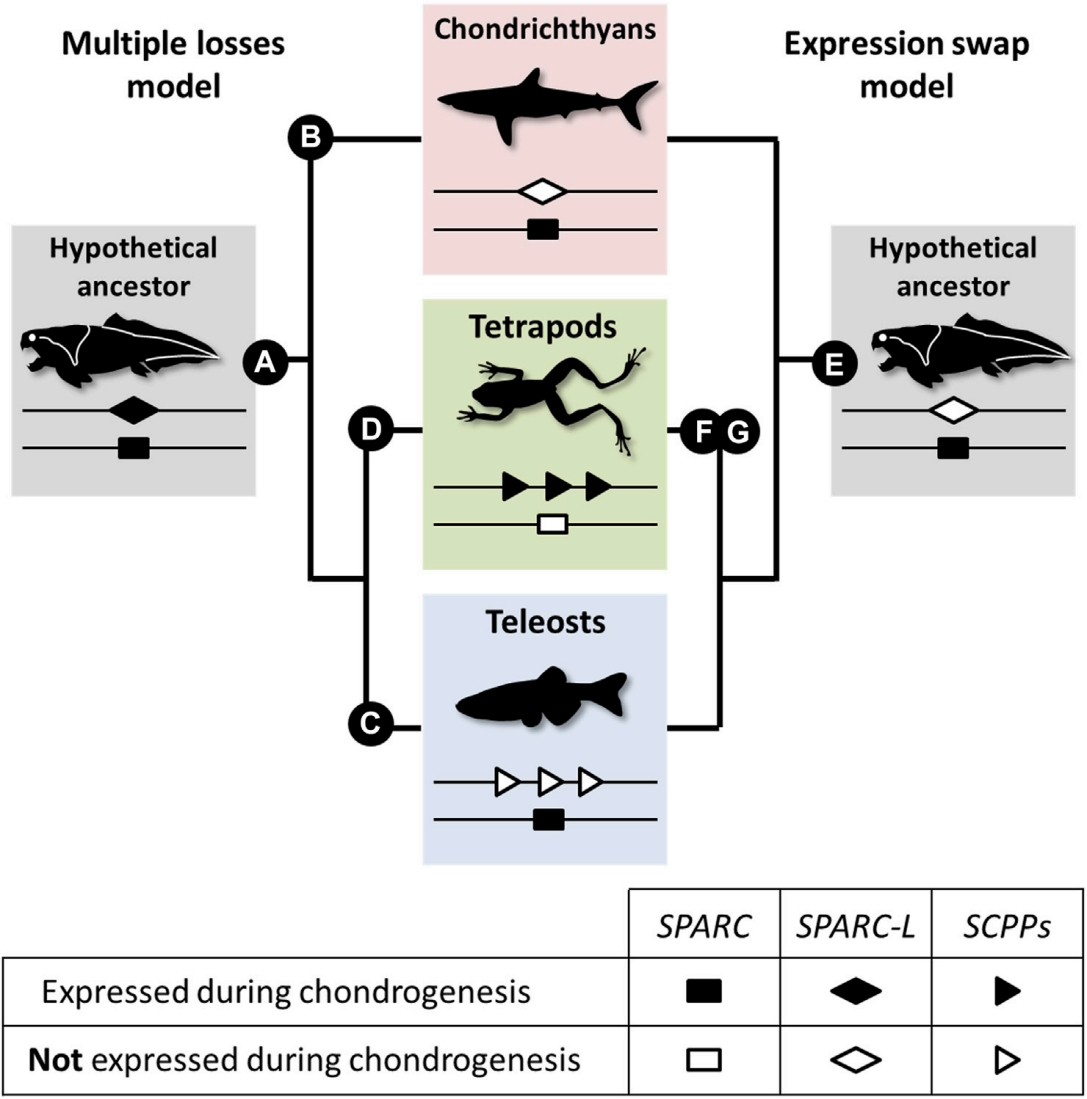
A model for the evolution of the *SPARC*/*SPARC-L*/*SCPP* cartilaginous expression in jawed vertebrates. Evolutionary changes are polarized onto vertebrate cladograms. According to the “multiple losses” model **(left)**, the mineralizing cartilage of the jawed vertebrate last common ancestor expressed both the *SPARC* and *SPARC-L* ancestral genes **(A)**. This expression was inherited by the *SCPP* members when they evolved through *SPARC-L* local duplications. Expression losses occurred at least three times independently in distinct lineages: *SPARC-L* expression was lost in chondrichthyans **(B)**, *SCPP* expression was lost in teleosts **(C)** and *SPARC* expression was lost in tetrapods **(D)**. According to the “expression swap” model **(right)**, the mineralizing cartilage of the jawed vertebrate last common ancestor only expressed *SPARC* **(E)**, a situation which remained unchanged in the chondrichthyan and teleost lineages. However, the tetrapod lineage experienced drastic regulatory changes at both loci, leading to the activation of the *SCPP* genes **(F)** and the repression of *SPARC* **(G)** in chondrocytes.

Our data suggest that chondrichthyans are more similar to teleosts than to tetrapods because the *Sc* mineralizing cartilage is *SPARC* positive and *SPARC-L* negative. Here, we propose two evolutionary models to explain these divergent expression patterns (see Figure 5 and Supplementary Figure S1). Both models are based on the assumption that *SPARC*, *SPARC-L*, and *SCPP* share some level of functional redundancy, at least during chondrogenesis, as suggested by the fact that both *SPARC* and *SCPP* proteins are extracellular transglutaminase substrates (Aeschlimann et al., 1995; Forsprecher et al., 2011) and bind calcium ions (Engel et al., 1987; Chen et al., 1992; Maurer et al., 1995; Klaning et al., 2014). The “multiple losses” model is reminiscent of the Duplication Degeneration Complementation (DDC) phenomenon (Force et al., 1999) and involves at least three independent changes which abrogated the cartilaginous expression of *SPARC*, of *SPARC-L* or of the *SCPP* genes in distinct lineages (Figure 5, left panel). The “expression swap” model involves two changes and implies that, in the tetrapod lineage, cartilaginous expression was gained for the *SCPP* genes and lost for *SPARC* (Figure 5, right panel). While the *SPARC*/*SPARC-L*/*SCPP* family exhibit a unique evolutionary trajectory (Bertrand et al., 2013; Enault et al., 2018), the “expression swap” model would be similar to the dynamic expression turnover observed between members of the *Keratin*, *Vitellogenin*, and *Globin* vertebrate families (Finn et al., 2009; Vandebergh and Bossuyt, 2012; Opazo et al., 2015). Both scenarios imply regulatory changes that switched off (“multiple losses model”) or on (“expression swap model”) several *SCPP* genes (Figure 5). From a regulatory perspective, the idea of coordinated change in the expression of tandemly located *SCPP* genes is consistent with the fact that *BSP* and *DMP1* are included within the same topological association domain in human chromatin (Krietenstein et al., 2020), and that multiple genes can be co-regulated by the same enhancer (reviewed in Zheng and Xie, 2019). To the best of our knowledge, current experimental evidence is not sufficient to discriminate between the two models shown in Figure 5. Hence, a detailed analysis of the expression and regulation of *SPARC*/*SPARC-L*/*SCPP* genes in chondrocyte from a broader array of chondrichthyan and osteichthyan species will be required to shed light on the genetic mechanisms involved in the evolution of cartilage mineralization that originated deep in the vertebrate lineage (Min and Janvier, 1998; Donoghue et al., 2006; Johanson et al., 2010; 2012).

## Data Availability

The raw data supporting the conclusion of this article will be made available by the authors, without undue reservation.
