# Supplementary figures and images for: Divergent Expression of SPARC, SPARC-L, and SCPP Genes During Jawed Vertebrate Cartilage Mineralization
Source: Front Genet. 2021 Nov 25;12:788346. doi: 10.3389/fgene.2021.788346 (PMC8656109; doi:10.3389/fgene.2021.788346)

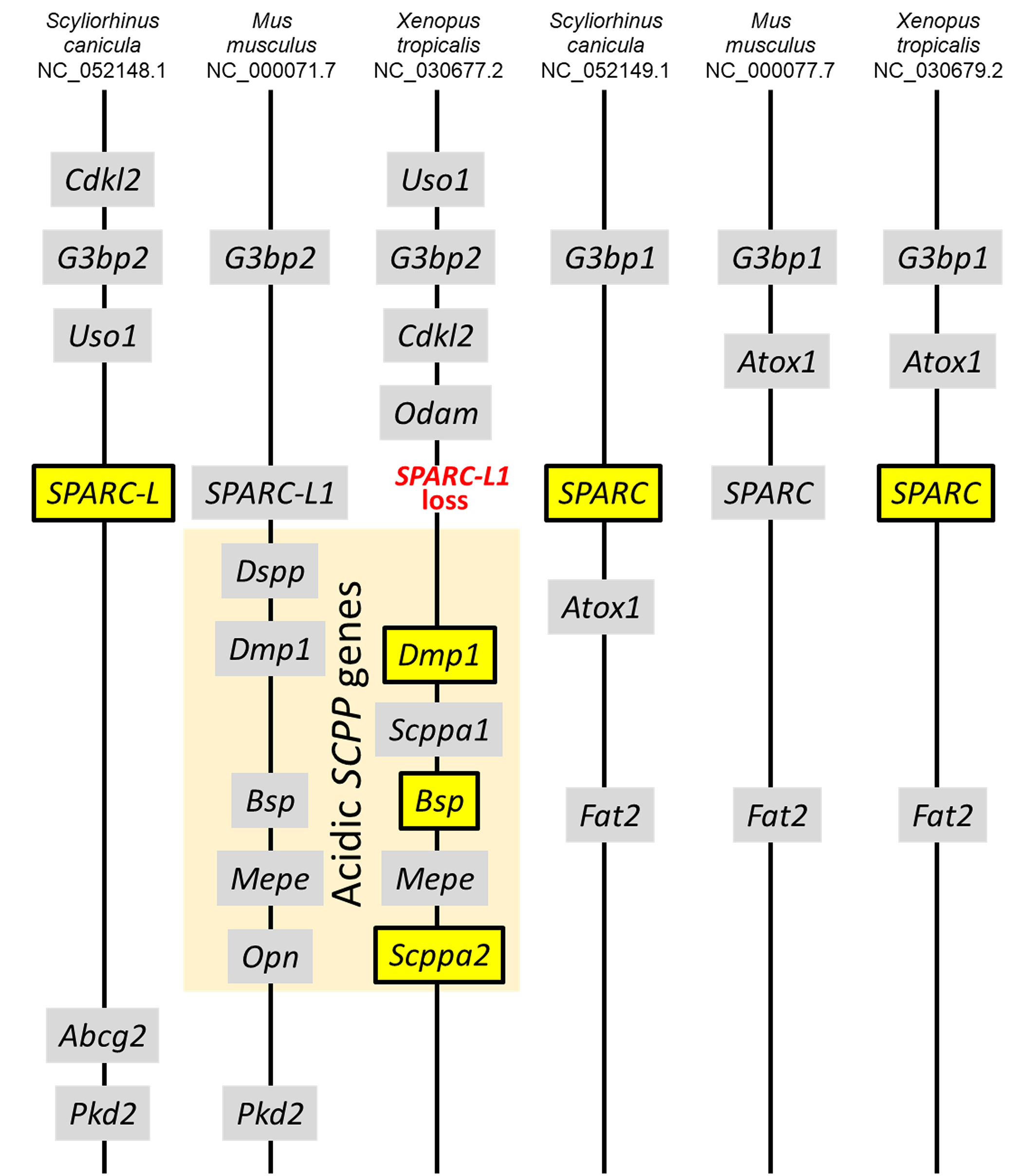

Supplement: Supplementary file 1 [file Image1.JPEG]
